# Deviation From Genetically Predicted BMI and All‐Cause Mortality: A Cohort Study in the UK Biobank

**DOI:** 10.1002/oby.70042

**Published:** 2025-10-02

**Authors:** Nuno R. Zilhao, Jie Zhang, Dorret I. Boomsma, Thorkild I. A. Sørensen, Christina C. Dahm

**Affiliations:** ^1^ Department of Public Health Aarhus University Aarhus Denmark; ^2^ Steno Diabetes Center Aarhus Aarhus University Hospital Aarhus Denmark; ^3^ Complex Trait Genetics, Center for Neurogenomics and Cognitive Research Vrije Universiteit Amsterdam Amsterdam the Netherlands; ^4^ Novo Nordisk Foundation Center for Basic Metabolic Research and Department of Public Health University of Copenhagen, and Centre for Childhood Health Copenhagen Denmark

## Abstract

**Objective:**

The relation between genetically predicted BMI (gBMI) and actual BMI may have health effects. This study examines the relationship between deviations from gBMI and all‐cause mortality in 208,146 UK Biobank participants.

**Methods:**

We derived gBMI from polygenic risk scores, with deviations calculated as the difference between observed and predicted BMI. Cox proportional hazards models are adjusted for confounders and current BMI.

**Results:**

Downward deviations (> 2 SD below gBMI) were associated with significantly increased mortality (HR: 1.25, 95% CI: 1.01–1.55), whereas upward deviations (> 2 SD above) showed no significant effect (HR: 1.10, 95% CI: 0.93–1.29). The mortality exhibited the known nonlinear J‐shaped association with observed BMI, here lowest at BMI ~22 kg/m^2^, but this nadir varied by genetic predisposition; thus, for individuals with high gBMI, lowest mortality occurred at higher observed BMI (24–26 kg/m^2^), while those with low or medium gBMI showed sharper increases in mortality at higher BMI.

**Conclusions:**

These findings highlight the possible importance of aligning current BMI to genetic predisposition, and future research should examine BMI deviations and their long‐term health effects. This perspective may inform personalized obesity management strategies to optimize health outcomes.

**Study Importance**

- What is already known?
  ○ BMI is a widely used measure for assessing health risk, and its association with mortality is known to be nonlinear.
  ○ Genetic factors substantially influence BMI, yet the consequences of deviations from an individual's genetically predicted BMI are not well understood.
- What does this study add?
  ○ This study demonstrates that deviations from genetically predicted BMI—whether above or below—are associated with increased mortality, independent of observed BMI.
  ○ It also shows that the relationship between BMI and mortality differs by level of genetic predisposition.
- How might these results change the direction of research or the focus of clinical practice?
  ○ Our findings suggest that individuals whose observed BMI aligns more closely with their genetically predicted BMI may have lower mortality risk.
  ○ This highlights the potential importance of considering genetic predisposition when interpreting BMI in both research and clinical settings.

## 1 Introduction

Obesity is a global epidemic, contributing to type 2 diabetes, cardiovascular disease, and fatty liver disease [1, 2, 3]. Not all individuals with obesity show metabolic dysregulation, inspiring the concept of “healthy obesity,” while some with normal or overweight body mass index (BMI) display metabolic disturbances [4, 5, 6].

In the UK Prospective Diabetes Study, one‐third of new type 2 diabetes cases occurred in individuals with BMI < 25 kg/m^2^ [5]. Weight loss can induce remission of type 2 diabetes and nonalcoholic fatty liver disease in individuals with both obesity and normal weight. These findings support the “Personal Fat Threshold” (PFAT) and “Lipotoxicity” hypotheses, proposing that fat storage capacity and distribution influence metabolic health [7, 8, 9].

The PFAT hypothesis proposes that individuals have a personal threshold for safe fat storage, beyond which excess fat accumulates in organs like the liver and muscle, causing metabolic dysfunction. This threshold varies, explaining why some remain metabolically healthy despite obesity, while others experience complications at lower adiposity. The Lipotoxicity hypothesis extends this by suggesting that exceeding the threshold leads to toxic fat accumulation, promoting inflammation and insulin resistance. These models align with the adipose expandability hypothesis, which suggests that limited safe fat storage capacity leads to adverse health outcomes when exceeded [10].

Although the genetic variants responsible for setting individual PFATs are unknown, the genetic influence on BMI is substantial, with heritability estimates ranging from 40% to 70% [11, 12]. Genome‐wide association studies (GWAS) have identified multiple variants linked to obesity, which are summarized as polygenic risk scores (PGS) [13, 14, 15]. PGS can estimate individuals' genetically predicted BMI (gBMI), hypothesized to reflect an individual‐specific optimal BMI level with a survival advantage in the genetic lineage.

Experimentally addressing whether the PFAT hypothesis explains part of the link between obesity and mortality is challenging due to the scale and duration of clinical trials. However, observational studies show stable weight across a wide BMI range is associated with lower mortality than weight gain or loss [16, 17]. Recent longitudinal studies have further emphasized the impact of gBMI on BMI trajectories over time [18]. They demonstrated that individuals whose observed BMI significantly deviates from their PGS‐predicted BMI tend to follow distinct weight gain patterns over the long term, highlighting the role of genetic predisposition in weight stability and the risk of obesity [18, 19].

Assuming that aligning one's BMI with gBMI confers a survival advantage, these studies may suggest that individuals whose weight remains stable in adulthood are living at or near their genetically determined BMI and thus at their PFATs. Conversely, deviations from gBMI reflect a mismatch between genetic predisposition and realized BMI. Positive deviations may indicate individuals who have exceeded their PFATs, whereas negative deviations may indicate individuals below their threshold.

Here, we aimed to explore the relationship between deviations from gBMI and mortality. Given the substantial genetic influence on BMI, the individual variability in fat storage capacity, and known health risks of reduced muscle mass, we hypothesized that deviations in either direction—upward or downward—from gBMI may reflect physiological mismatch or dysregulation and would be associated with increased mortality compared to little or no deviation. This study tests this hypothesis by analyzing the association between BMI deviations and all‐cause mortality in a large cohort from the UK Biobank (UKB).

## 2 Methods

### 2.1 Study Population

This study analyzed data from UKB, a large‐scale prospective cohort consisting of approximately 502,490 individuals aged 37–73 years, recruited between 2006 and 2010. Participants were recruited using a nonprobability sampling design through mailed invitations to individuals registered with the UK National Health Service. Approximately 5% of those invited chose to participate [20, 21]. The recruitment, participant demographics, and quality control processes are described in prior studies [22]. To ensure only unrelated individuals were included in the analysis, we used the UKB Data Field 22,020, restricting to a maximal unrelated subset (up to 3rd degree), excluding sex discordance or outlier status for missingness or heterozygosity [23]. We removed individuals with missing data on BMI, sex, age, and mortality information, as well as individuals with outlier values for BMI, defined as 3 standard deviations (SD) above or below the mean. Additionally, we excluded individuals with key prediagnosed conditions to avoid potential biases in the association between BMI deviation and mortality. Exclusions encompassed individuals with known diabetes (self‐reported or doctor‐diagnosed) and cardiovascular diseases such as heart attack, angina, stroke, and hypertension, alongside those with cancer diagnoses and those using medications for diabetes, hypertension, or hyperlipidemia at baseline (e.g., insulin, metformin, statins, antihypertensives), given their potential influence on both BMI and mortality.

### 2.2 BMI Polygenic Risk Score Calculation

In our study, the BMI‐PGS employed was derived from the work of Thompson et al. where the authors calculated and released polygenic scores on 28 diseases and eight quantitative traits [24]. Here, BMI‐PGS was extracted from Standard PGS (Category 301), which was created through the meta‐analysis of various external GWAS sources, including those listed in Tables S1 and S2 of Thompson et al. [23], for all individuals in UKB. For a comprehensive description of how the BMI‐PGS was calculated, please refer to Thompson et al. [24].

### 2.3 Deviation From gBMI

Height and weight were measured using a Seca 202 stadiometer and a Tanita BC‐418 body composition analyzer, respectively, recorded at baseline. BMI was calculated as weight (in kilograms) divided by height (in meters squared) at baseline. gBMI was calculated using a linear regression model that included the BMI‐PGS, adjusted for age and sex. The residuals of this model are mathematically equivalent to subtracting observed BMI from predicted BMI with a positive residual indicating that the observed BMI is higher and a negative value that the observed BMI is lower than the predicted BMI. These deviations of the residuals were further categorized based on their magnitude, with individuals classified as “persons with deviant BMI” if their observed BMI deviated by more than 2 SD from the population mean.

### 2.4 Mortality Outcome Data

Mortality data was obtained through linkage to national death registries. Data consisted of date of death, age of death, and primary cause of death using International Classification of Diseases (ICD)‐10 codes for all deaths from 2006‐05‐10 to 2022‐12‐19. Individuals not registered as deceased by 2022‐12‐19 were presumed to be alive.

### 2.5 Covariates

Age, sex, socioeconomic status, smoking, and physical activity were considered confounders in analyses of BMI deviation and mortality. Age and sex were self‐reported at recruitment. Sex was confirmed using genetic data, and mismatches were removed. Socioeconomic status was assessed by the Townsend Deprivation Score, which is a measure of area‐level socioeconomic deprivation, and annual household income. Townsend Deprivation Score incorporates unemployment rates, car ownership, household overcrowding, and owner occupation rates within the participants' postal areas, with higher scores indicating greater deprivation. Annual household income was self‐reported at baseline and used to categorize participants into five income brackets: < £18,000; £18,000 to £30,999; £31,000 to £51,999; £52,000 to £100,000; and > £100,000. Physical activity was quantified based on participants' self‐reported weekly engagement in light, moderate, and vigorous activities, summed into a composite weekly activity score. Smoking status was categorized into “never,” “former,” and “current” (for further details, please refer to online Supporting Information).

### 2.6 Cox Proportional Hazards Models

To benchmark prior findings, we examined the relationship between observed BMI and all‐cause mortality, modeling observed BMI as a continuous variable using natural splines with four degrees of freedom (4 df). Cox proportional hazards models adjusted for covariates were used to estimate hazard ratios (HRs) with 95% confidence intervals (CIs) relative to the median observed BMI. Age served as the primary time‐scale variable in all Cox proportional hazards models, with entry time defined as age at baseline and exit time defined as age at death or age at the end of follow‐up (whichever occurred first). Mortality status was coded as 1 if the participant had died during follow‐up and 0 otherwise. The models were adjusted for sex, Townsend Deprivation Score, household income, smoking, and physical activity.

To assess the association between deviation from gBMI and all‐cause mortality, we modeled BMI deviations as natural splines in Cox proportional hazards regression models adjusted for the same covariates. Results were expressed as HRs. The reference point was zero deviation, and natural splines were defined (4 df) to account for potential nonlinear associations between deviation from gBMI and all‐cause mortality. The number of knots for the splines was determined by the Bayesian information criterion (BIC), selecting the model with the lowest BIC value. Knots were placed at quantiles of the BMI deviation distribution.

In addition, the association between gBMI and mortality was evaluated to assess its independent effect, separate from deviations. gBMI was modeled as a continuous variable using natural splines (4 df), adjusted for the same covariates. HRs were calculated relative to the median gBMI, benchmarking the hypothesis that gBMI alone does not drive mortality risk.

The proportional hazards assumption was tested using Schoenfeld residuals, and all covariates were found to satisfy this assumption. The significance of the spline model was evaluated by comparing it to a linear model using a likelihood ratio test.

### 2.7 Subgroup Analyses

We repeated the analyses stratified by sex, age (categorized into three groups: 37–49, 50–59, and 60–75 years), smoking status (never, former, and current smokers at the time of BMI assessment), and BMI categories (underweight [< 18.5 kg/m^2^], normal weight [18.5–24.9 kg/m^2^], overweight [25.0–29.9 kg/m^2^], and obesity [≥ 30 kg/m^2^], based on WHO classifications) to assess whether associations differed across these factors.

### 2.8 Analysis of Extreme Deviation From gBMI and Mortality

We further examined the impact of extreme deviations from gBMI on mortality risk in Cox proportional hazards models adjusted for age (used as the time‐scale variable), sex, Townsend Deprivation Score, household income, smoking, physical activity, and observed BMI, modeled using natural splines (4 df) to capture potential nonlinear associations. The key predictor was a categorical variable indicating > 2 SD BMI deviations—upward (observed BMI > predicted BMI) or downward (observed BMI < predicted BMI). Separate analyses were conducted for individuals in the observed normal BMI range (18.5–24.9 kg/m^2^) and across the full observed BMI spectrum.

### 2.9 Tertile Analysis of gBMI

To assess observed BMI–mortality relationships across genetic predisposition levels, we stratified participants into tertiles of gBMI (low, medium, high). Cox models with natural splines (4 df) fitted were fitted to examine the association between observed BMI and all‐cause mortality separately within each tertile of gBMI. Models were adjusted for age (as the primary time scale), sex, Townsend Deprivation Score, household income, smoking, and physical activity. Observed BMI was modeled continuously using natural splines.

Analyses were conducted using R in the UKB Research Analysis Platform.

### 2.10 Ethical Considerations

The study was conducted in accordance with the ethical standards of the institutional research committee and with the 1964 Helsinki Declaration and its later amendments or comparable ethical standards. The UKB adhered to ethical guidelines approved by the NHS National Research Ethics Service North West (REC reference: 11/NW/0382).

## 3 Results

After applying quality control parameters for phenotypic and genetic data, 335,308 White British ancestry participants with valid BMI, genetic, and survival data were included. To reduce population stratification and ensure genetic comparability, we restricted the analysis to individuals of White British ancestry, as defined by genetic principal components and self‐reported ethnicity. Further exclusions based on kinship, preexisting conditions, and outliers resulted in a study population of 208,442 individuals. Participants had a mean age of 54.6 years (SD 8.1); 54.3 (8.2) for males and 54.8 (8.0) for females. The mean BMI was 26.4 (SD 4.0); 26.9 (3.6) for males and 26.0 (4.3) for females (Table 1). By the end of follow‐up (median follow‐up: 7 years), 5771 had died, primarily due to cancers and cardiovascular diseases (Table S1).

**TABLE 1:** Demographic and clinical characteristics of UK Biobank participants by gender.

Variable	Female (*N* = 120,725)	Male (*N* = 87,421)
Age (years)	54.83 (8.00)	54.27 (8.23)
Age 37–49, *n* (%)	25,552 (21.17%)	19,574 (22.39%)
Age 50–59, *n* (%)	52,717 (43.67%)	37,221 (42.58%)
Age 60–75, *n* (%)	42,456 (35.16%)	30,626 (35.03%)
BMI (kg/m^2^)	26.01 (4.33)	26.86 (3.64)
Body fat (%)	35.28 (6.65)	23.81 (5.49)
Smoking pack years	18.41 (14.15)	22.42 (18.06)
Smokers, *n* (%)	8012 (6.63%)	8042 (9.19%)
Nonsmokers, *n* (%)	112,575 (93.11%)	79,229 (90.51%)
Townsend Index	−1.44 (3.00)	−1.36 (3.11)
Income (£)	4.43 (2.10)	4.19 (1.94)
Physical Activity (days)	5.42 (3.57)	5.82 (3.82)

*Note*: Continuous variables are reported as mean ± SD, while categorical variables, such as smoking status, are presented as counts with corresponding percentages.

The BMI‐PGS explained 6.3% of BMI variance. For every unit increase in Standard PGS for BMI, the observed BMI was approximately 1.05 units higher. As expected, the range of observed BMI was greater than that of gBMI (Figure S1). Of the 208,146 participants, 9944 had BMI values deviating by more than 2 SD from their gBMI, with 1390 below and 8554 above predicted levels. To clarify the relationship between observed BMI and BMI deviations across the full sample, we plotted the mean deviation within 1‐unit bins of observed BMI (Figure S2). A strong linear pattern emerged: lower observed BMI showed negative deviation, while higher BMI showed positive deviation. This asymmetry suggests upward deviation is more common, likely reflecting environmental or behavioral influences.

**FIGURE 1:**
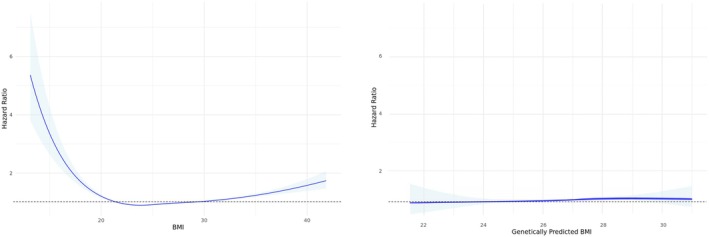
Predicted hazard ratio as a function of BMI deviation. Natural splines with four degrees of freedom were employed to model the nonlinear effects of observed BMI (left panel) and gBMI (right panel) on all‐cause mortality. The solid lines represent the predicted hazard ratios across the respective BMI ranges, while the shaded areas indicate the 95% CI. Both models were adjusted for sex, age at recruitment, total physical activity, Townsend Deprivation Index, aggregated income, and smoking category. The dotted horizontal lines indicate a hazard ratio of 1, highlighting the BMI levels at which mortality risk is equal to the reference group. [Color figure can be viewed at wileyonlinelibrary.com]

We first examined the relationship between observed BMI and all‐cause mortality. A spline Cox model provided a significantly better fit than a linear model (*p* < 2.2e‐16), indicating a non‐linear relationship with lowest mortality at 23.8 kg/m^2^. HRs increased below and above the reference group centered around median BMI. We next examined the relationship between gBMI and mortality. The HR curve was flat, suggesting no strong association (Figure 1).

**FIGURE 2:**
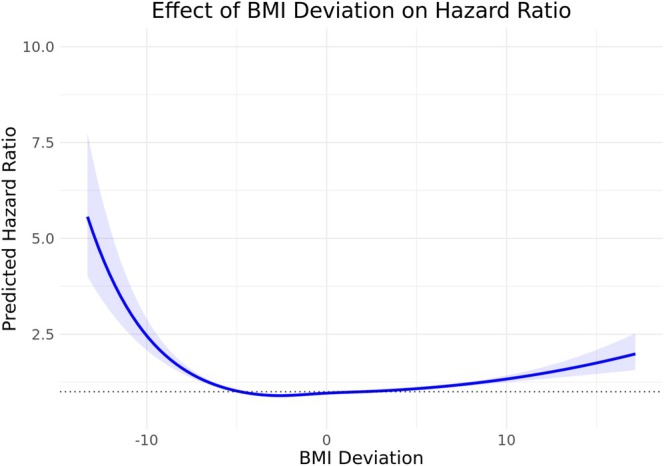
Predicted hazard ratio as a function of BMI deviation. Natural splines with four degrees of freedom were employed to model the nonlinear association between BMI deviation and all‐cause mortality. The solid line represents the predicted hazard ratios across the range of BMI deviation, while the shaded area indicates the 95% CI. The model was adjusted for sex, age at recruitment, total physical activity, Townsend Deprivation Index, aggregated income, and smoking category. The dotted horizontal line indicates a hazard ratio of 1, highlighting the BMI deviation at which mortality risk is equal to the reference group. [Color figure can be viewed at wileyonlinelibrary.com]

**FIGURE 3:**
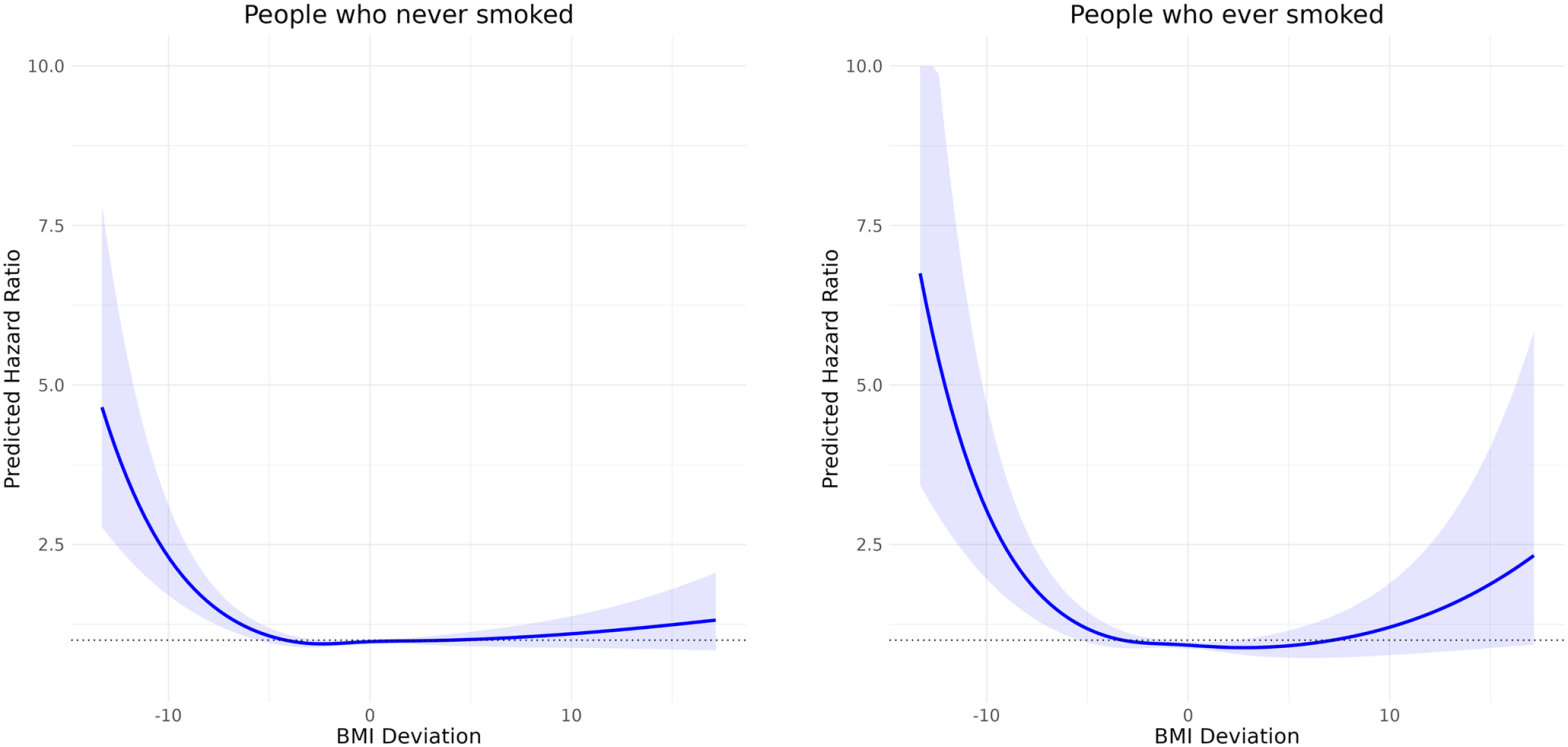
Predicted hazard ratio as a function of BMI deviation, stratified by smoking status. Natural splines with four degrees of freedom were used to model the nonlinear effects of BMI deviation on all‐cause mortality, shown separately for individuals who had never smoked (left panel) and those who had ever smoked (right panel). The solid lines indicate the predicted hazard ratios, with shaded areas showing 95% CI. Models were adjusted for sex, age at recruitment, total physical activity, Townsend Deprivation Index, and income. The dotted horizontal line indicates a hazard ratio of 1. [Color figure can be viewed at wileyonlinelibrary.com]

We then assessed BMI deviation and all‐cause mortality. Deviation was nonlinearly associated with mortality (Figure 2). The association held for both smokers and never‐smokers, though it was attenuated in the latter (Figure 3). Associations were similar by age but varied slightly across WHO‐defined BMI categories (Figures S3 and S4).

To explore extreme deviation and mortality risk, we first examined individuals with observed BMI in the normal range (18.5–24.9 kg/m^2^), then extended to the full BMI spectrum. As expected, fewer individuals in this range had deviations > 2 SD. Among individuals with normal‐range BMI, no significant mortality association was found for BMI deviations ≥ 2 SD (HR: 1.04, 95% CI: 0.95–1.14), after adjustment for BMI, age, sex, deprivation, income, smoking, and physical activity. In the full BMI spectrum, participants with > 2 SD BMI deviations had 15% higher mortality risk vs. those within 2 SD (HR: 1.15, 95% CI: 1.01–1.31), adjusting for BMI and covariates. Negative deviation (observed BMI < predicted BMI) was linked to higher mortality (HR: 1.25, 95% CI: 1.01–1.55), while positive deviation showed no significant risk (HR: 1.10, 95% CI: 0.93–1.29).

**FIGURE 4:**
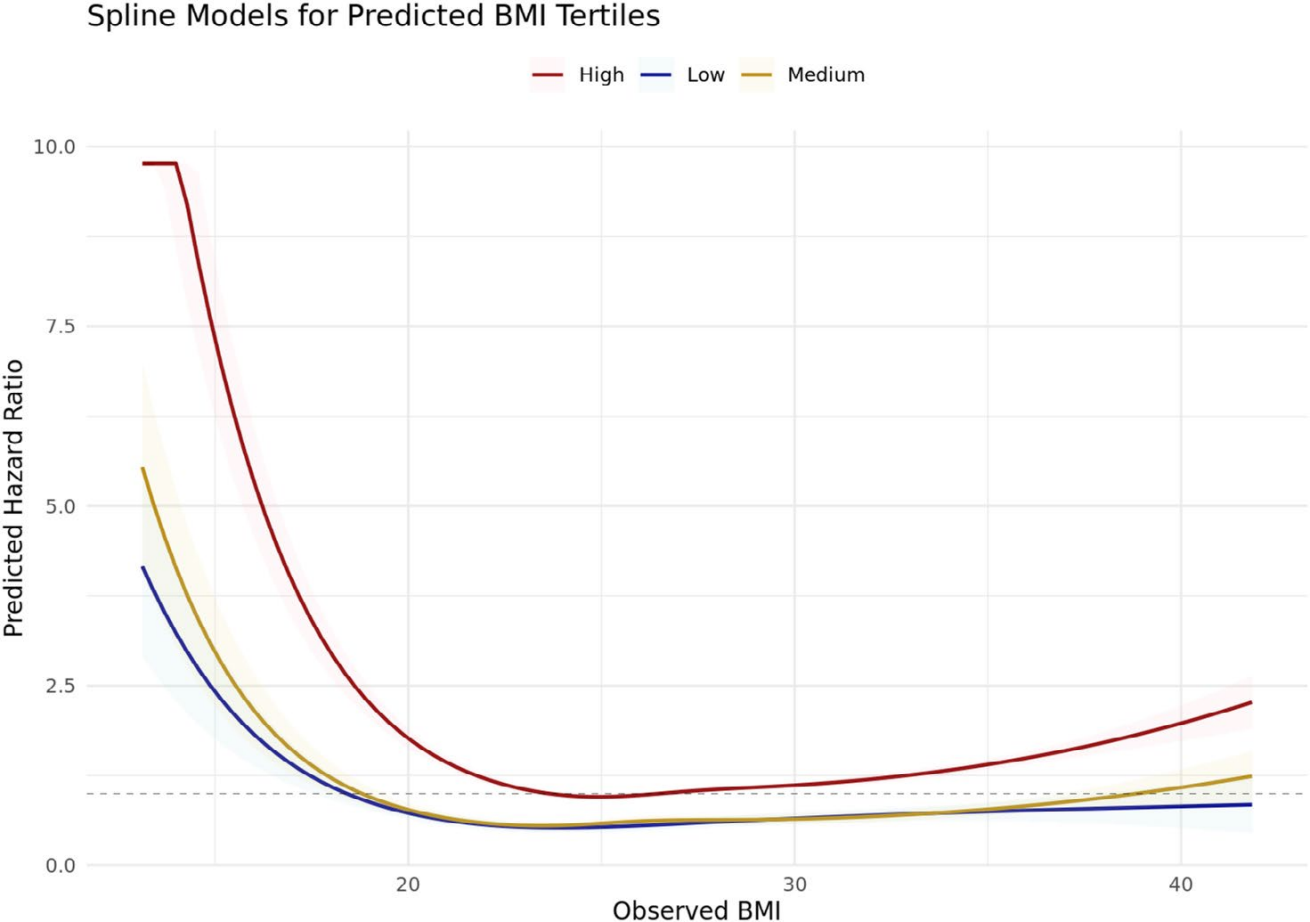
Observed BMI and mortality risk by tertiles of genetically predicted BMI. Natural splines with four degrees of freedom were used to model the association between observed BMI and all‐cause mortality, stratified by tertile of gBMI: Low (left), medium (center), and high (right). Solid lines represent hazard ratios across BMI values; shaded areas indicate 95% CI. Models were adjusted for age, sex, total physical activity, Townsend Deprivation Index, aggregated income, and smoking category. The dotted horizontal line represents the reference hazard ratio of 1.0. [Color figure can be viewed at wileyonlinelibrary.com]

To assess how genetic predisposition modifies BMI‐mortality associations, we stratified participants by tertiles of gBMI. Observed BMI–mortality associations varied across gBMI tertiles. Across tertiles, observed BMI had a J‐shaped relationship with mortality, with the lowest risk at 22–25 kg/m^2^. However, the magnitude and shape of associations differed. In low and medium tertiles, the lowest mortality occurred near 22 kg/m^2^. For the high tertile, it shifted to 24–26 kg/m^2^. In the high tertile, mortality rose more gradually at higher BMI (Figure 4).

## 4 Discussion

We examined how deviation between observed BMI and gBMI associates with mortality. We found that higher observed BMI is more strongly linked to mortality in individuals with low or medium gBMI, suggesting lower tolerance to excess weight. We further showed that the magnitude and direction of deviation from gBMI were significantly and nonlinearly associated with mortality. Alignment with genetic predisposition may reduce mortality risk by maintaining genetically optimal adiposity and metabolic health. Our findings underscore the importance of minimizing the mismatch between genetic predisposition and realized BMI.

Although BMI does not precisely capture individual differences in body composition and adiposity, greater deviation from genetic predisposition—upward or downward—may reflect physiological or behavioral factors such as excess fat, low muscle mass, or illness‐related weight changes. BMI is not tissue‐specific and cannot distinguish fat mass, lean mass, or distribution—factors that may influence health and mortality. While body composition measures could illuminate compartment‐specific contributors to BMI deviations, they cannot directly assess fat storage thresholds. The role of deviation between observed BMI and gBMI in morbidity and mortality may be further elucidated by decomposing BMI and its deviation into body composition and body shape in future studies. The PFAT hypothesis posits genetically determined limits to safe fat storage, beyond which metabolic dysregulation occurs. Exceeding this threshold leads to fat accumulation in nonadipose tissues, causing metabolic disturbances [5, 7, 8, 25]. Supporting evidence shows modest weight loss improves metabolic health by reducing liver and pancreatic fat, with reductions in hepatic fat and improved insulin sensitivity leading to diabetes remission [6, 7]. While we could not directly test the PFAT hypothesis—due to unknown variants—our results do not support that greater positive deviation is linked to higher mortality at higher levels of the BMI distribution. Future studies using fat distribution and ectopic fat data may clarify links to excess fat deposition, although direct testing of the PFAT hypothesis will require detailed knowledge of functional variants.

Observed BMI remained a strong mortality predictor, showing the well‐known nonlinear risk increase at both BMI extremes. This relationship varied across tertiles of gBMI. The lowest mortality occurred at ~22 kg/m^2^ for low/medium tertiles and ~25 kg/m^2^ for the high tertile. The magnitude and slope of this association also differed by gBMI tertile. In individuals with low or medium gBMI, mortality risk increased sharply with higher observed BMI, suggesting lower physiological tolerance to excess weight. Conversely, in individuals with high gBMI, mortality risks rose more gradually with increasing observed BMI, indicating a degree of genetic tolerance to higher weight. As hypothesized, gBMI alone showed no strong mortality association. This lack of a direct gBMI–mortality association is consistent with Mendelian randomization findings in UKB that reported a hazard ratio of 1.03 (95% CI: 0.99–1.07) for all‐cause mortality per 1 kg/m^2^ gBMI in 335,308 participants, using 77 genome‐wide significant SNPs [26]. In the present study as well as in several other large cohort studies, the association of observed baseline BMI within the range of gBMI with mortality is very weak. The elevated mortality emerges at BMI levels below and above this range, hence making the typical J‐ or U‐shaped BMI‐mortality association. This supports our conceptualization of gBMI as a neutral reference point—representing genetic liability but not directly encoding health risk. However, we acknowledge that the absence of a gBMI–mortality association does not, in itself, validate gBMI as a physiologically meaningful set point. Rather, it emphasizes that deviation is only interpretable as a departure from a so far undetermined gBMI outside the current range insofar as the current gBMI is assumed to reflect the inherited predisposition also for people outside that range. The relatively flat hazard ratio suggests genetic predisposition alone does not drive mortality risk. This weak association may also be attenuated by characteristics of the UKB cohort, including birth cohort trends toward higher BMI in more recent decades, which may obscure genetic effects over time [27]. This underscores the role of BMI deviations—differences between observed BMI and gBMI—as key mortality risk factors. This supports our core hypothesis: that it is not gBMI itself, but deviation—a behavioral, environmental, or pathological mismatch—that more strongly drives mortality risk.

We also observed higher mortality with greater negative deviation from gBMI. This may reflect critically low levels of muscle and skeletal mass, unmeasured environmental, or preclinical disease processes.

One prior study examined BMI deviations in relation to disease outcomes and found that individuals whose observed BMI was significantly higher than their gBMI had a greater risk of developing type 2 diabetes [28]. A recent study by Berntzen et al. found that individuals whose BMI deviated significantly from their genetically predicted levels exhibited distinct weight gain patterns over a 36‐year period [18]. A consistent finding is that stable weight is linked to lower mortality than weight loss or gain [29, 30]. Studies have shown that normal‐weight individuals reporting weight loss had higher mortality compared to those with stable weight [19]. Further support comes from findings that weight stability, especially when aligned with a lean body shape, is associated with lower mortality [31].

gBMI is a concept theoretically present from conception, while observed BMI changes over the life course supposedly are driven by changes in other influences. Deviation from gBMI highlights the degree of mismatch between genetic predisposition and environmental, behavioral, or biological factors, emphasizing how these factors influence mortality risk. As we were unable to determine when, or whether, participants' observed BMI had aligned with their gBMI, our study is unable to elucidate the long‐term relationship between BMI deviations and weight stability. However, our findings emphasize that even within the normal BMI range, significant deviation from gBMI is associated with health risks, indicating that genetic predisposition may offer additional perspectives on BMI‐related health outcomes. Further studies linking gBMI to metabolic biomarkers or physiological traits may help validate its use as a meaningful reference point. In summary, our findings suggest that it is not gBMI itself, but the deviation from it—especially in magnitude and direction—that more strongly associates with mortality risk. Future studies could examine how BMI deviation changes relate to morbidity and mortality. Longitudinal studies with diverse populations could provide further insights into the long‐term effects of BMI deviations on health outcomes. Exploring mechanisms behind these associations may inform better public health strategies.

One of the strengths of our study is the use of a large, well‐characterized cohort from UKB, which provides robust genetic and phenotypic data. The PGS calculations were conducted independently of UKB data, reducing potential bias from the discovery dataset when applying these scores to our study cohort. Height and weight were measured objectively at study centers, reducing measurement error and information bias. Although BMI was measured at multiple timepoints in UKB, we used only the baseline measurement to maximize sample size and statistical power. This approach is common in large cohort studies, but it limits our ability to account for weight changes during follow‐up. As a result, dynamic associations between weight trajectories and mortality risk may be underestimated or obscured. Future studies with repeated BMI measures may better model long‐term outcomes. We also excluded individuals on medications for blood pressure, cholesterol, or diabetes to minimize confounding from treatment effects on both BMI and mortality risk.

This approach may aid research or risk stratification by using genetic expectations to flag physiological mismatch.

However, our study has limitations. The participation rate of UKB was approximately 5% of those invited, which may introduce selection bias if associations between BMI deviation and mortality differ between participants and the general population. Yet, recent evaluations suggest this bias has limited impact on internal associations in hypothesis‐testing contexts. In particular, weighting analyses showed minimal influence on dimensionality estimation and large‐scale association testing, although prevalence and effect size estimates may be attenuated [32]. In our study, any selection‐related bias is likely nondifferential with respect to BMI deviation and mortality, and may thus bias our estimates toward the null. Additionally, the exclusion of individuals with preexisting cardio‐metabolic diseases may have resulted in a healthier subset of individuals with obesity, potentially underestimating the true mortality risk associated with excess weight. This within‐cohort selection process, whereby individuals with obesity who have survived without complications are overrepresented, could attenuate the observed associations or even create opposite, seemingly paradoxical patterns. While the BMI‐PGS used in our analyses allowed for a larger sample size, it explained only 6.3% of the variance in BMI. This trade‐off between predictive accuracy and sample size may have influenced the precision of our gBMI estimates. As a result, some persons with deviant BMI may not truly be categorized as such if more comprehensive PGS—accounting for the full genetic contribution to BMI, including X‐chromosomal and non‐SNP variants—were available.

Additionally, we observed asymmetry in BMI deviations, with a larger number of individuals having observed BMI above their genetically predicted levels. This likely reflects real‐world environmental and behavioral influences—such as the prevalence of obesogenic exposures—rather than poor calibration of the PGS. The BMI‐PGS used in our study was developed independently of UKB to avoid overfitting and currently explains 6.3% of BMI variance in this sample. While this reflects only a portion of the total genetic contribution to BMI, it highlights that observed BMI captures substantial nongenetic variation. Skewness in the distribution of BMI deviations may thus be informative in itself, capturing population‐level mismatches between genetic predisposition and realized phenotype—potentially relevant for public health. Clearly, the range of PRS‐predicted BMI—the gBMI—is narrower than the observed BMI both in the lower and upper tail (Figure S1). In particular, the right‐sided skewness is manifested by BMI values above the upper range of the currently available gBMI. Future research might address how much of the range outside this gBMI is attributable to poly‐ and/or monogenetic effects and environmental influences pushing the people away from their extended predicted BMI. PRS trained and validated entirely within UKB now explain up to 17% of BMI variance [33]. While our present analysis used a BMI‐PGS explaining 6.3% of variance, these advances illustrate that future improvements in deviation estimation are feasible. Moreover, environmental or cohort‐level differences—such as selection bias, measurement error, or variation in obesogenic exposure—may also contribute to PRS miscalibration. However, the relative similarity in environmental profiles between the discovery cohorts and UKB may mitigate more extreme calibration errors within UKB. Nonetheless, generalizability remains limited, and the deviation metric should be interpreted cautiously as a model‐based construct rather than a direct biomarker. Our observed J‐shaped relationship between BMI and mortality was slightly less pronounced at higher BMI levels compared to some prior studies. This likely reflects the unique characteristics of the UKB cohort, which is known for its “healthy volunteer effect”: participants with extreme BMI or severe comorbidities may be underrepresented, attenuating observed high‐BMI mortality risk. Future studies should consider longitudinal designs that account for the life‐course impact of BMI deviations, including earlier metabolic risk development and survival bias. As our study included only individuals of White British ancestry, the findings may not generalize to other ancestry groups. Future research should assess whether associations between BMI deviation and mortality differ across ancestral backgrounds. Replication in independent cohorts could strengthen generalizability and clarify whether findings are consistent across populations. Finally, we did not perform cause‐specific mortality analyses, which may have clarified whether BMI deviation–mortality associations differ by cause of death. Given that cancer and cardiovascular disease account for most deaths in the cohort (Table S1), this should be explored in future studies. These limitations inform our study's interpretation and generalizability.

## 5 Conclusion

To conclude, our study demonstrates that while gBMI appears unassociated with mortality, observed deviations in BMI from gBMI are associated with mortality. While these findings contribute to a better understanding of BMI‐related health risks, further research is needed to determine whether aligning observed BMI with genetic predisposition has clinical relevance. These results underscore the complex relationship between genetics, obesity, and mortality, highlighting the importance of individual variability when assessing BMI‐related health risks.

## Conflicts of Interest

The authors declare no conflicts of interest.

## Supporting information


**Data S1:** oby70042‐sup‐0001‐supinfo.docx.

## Data Availability

This research has been conducted using the UK Biobank Resource under Application Number 81520. Access to the data is available upon application to the UK Biobank (https://www.ukbiobank.ac.uk/enable‐your‐research).
