# Supplementary material for: Deviation From Genetically Predicted BMI and All‐Cause Mortality: A Cohort Study in the UK Biobank
Source: Obesity (Silver Spring). 2025 Oct 2;34(1):228–36. doi: 10.1002/oby.70042 (PMC12724034; doi:10.1002/oby.70042)
Supplement: Supplementary file 1 — Data S1: oby70042‐sup‐0001‐supinfo.docx. [file OBY-34-228-s001.docx]

# Deviation from Genetically Predicted BMI and All-Cause Mortality: a cohort study in the UK Biobank

Nuno R. Zilhao^1^, Jie Zhang^2^, Dorret I. Boomsma^3^, Thorkild IA. Sørensen^4^, Christina C. Dahm^1^.

^1^ *Department of Public Health, Aarhus University, Bartholins Alle 2, DK-8000 Aarhus C, Denmark.*^2^ *Steno Diabetes Center Aarhus, Aarhus University Hospital*

^3^ *Dept Biological Psychology, Vrije Universiteit* *Amsterdam, Van der Boechorststraat 7-9, 1081 BT, Amsterdam, The Netherlands.*

^4^ *Novo Nordisk Foundation Center for Basic Metabolic Research and Department of Public Health, University of Copenhagen, and Centre for Childhood Health, Copenhagen, DK.*

# Abstract

# Objective: The relation between genetically predicted Body Mass Index (gBMI) and actual BMI may have health effects. This study examines the relationship between deviations from gBMI and all-cause mortality in 208,146 UK Biobank participants. Methods: We derived gBMI from polygenic risk scores, with deviations calculated as the difference between observed and predicted BMI. Cox proportional hazards models adjusted for confounders and current BMI. Results: Downward deviations (>2 SD below gBMI) were associated with significantly increased mortality (HR: 1.25, 95% CI: 1.01–1.55), whereas upward deviations (>2 SD above) showed no significant effect (HR: 1.10, 95% CI: 0.93–1.29). The mortality exhibited the known non-linear J-shaped association with observed BMI, here lowest at BMI ~22 kg/m², but this nadir varied by genetic predisposition; thus, for individuals with high gBMI, lowest mortality occurred at higher observed BMI (24–26 kg/m²), while those with low or medium predicted BMI showed sharper increases in mortality at higher BMI. Conclusions: These findings highlight the possible importance of aligning current BMI to genetic predisposition, and future research should examine BMI deviations and their long-term health effects. This perspective may inform personalized obesity management strategies to optimize health outcomes.

# Supplemental Information

## Supplementary Figures


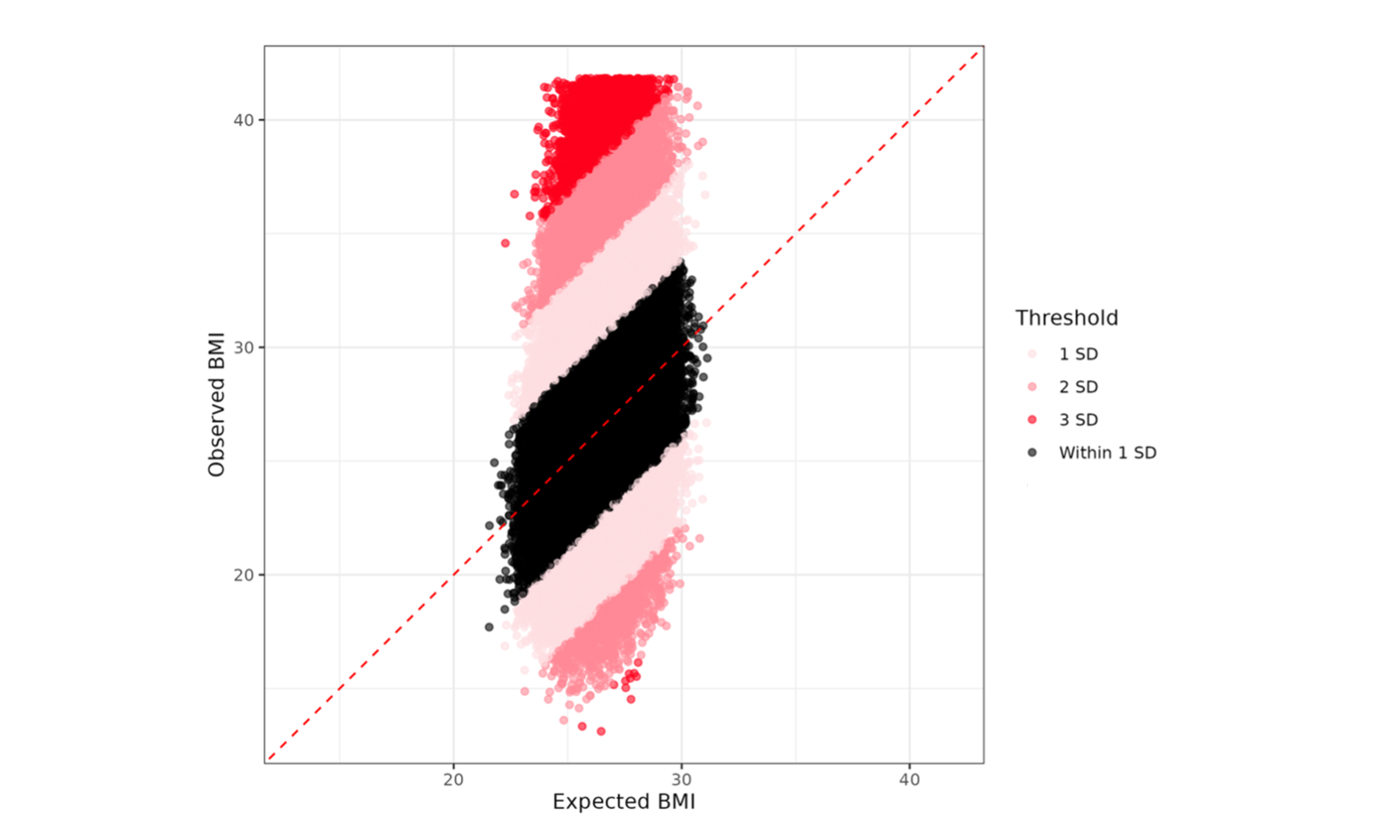


Supplementary Figure S1. Comparison of Observed vs. PRS-Predicted BMI across the study population. Scatter plot illustrating the relationship between PGS-predicted BMI and observed BMI. Each point represents an individual, with PGS-predicted BMI on the x-axis and observed BMI on the y-axis.


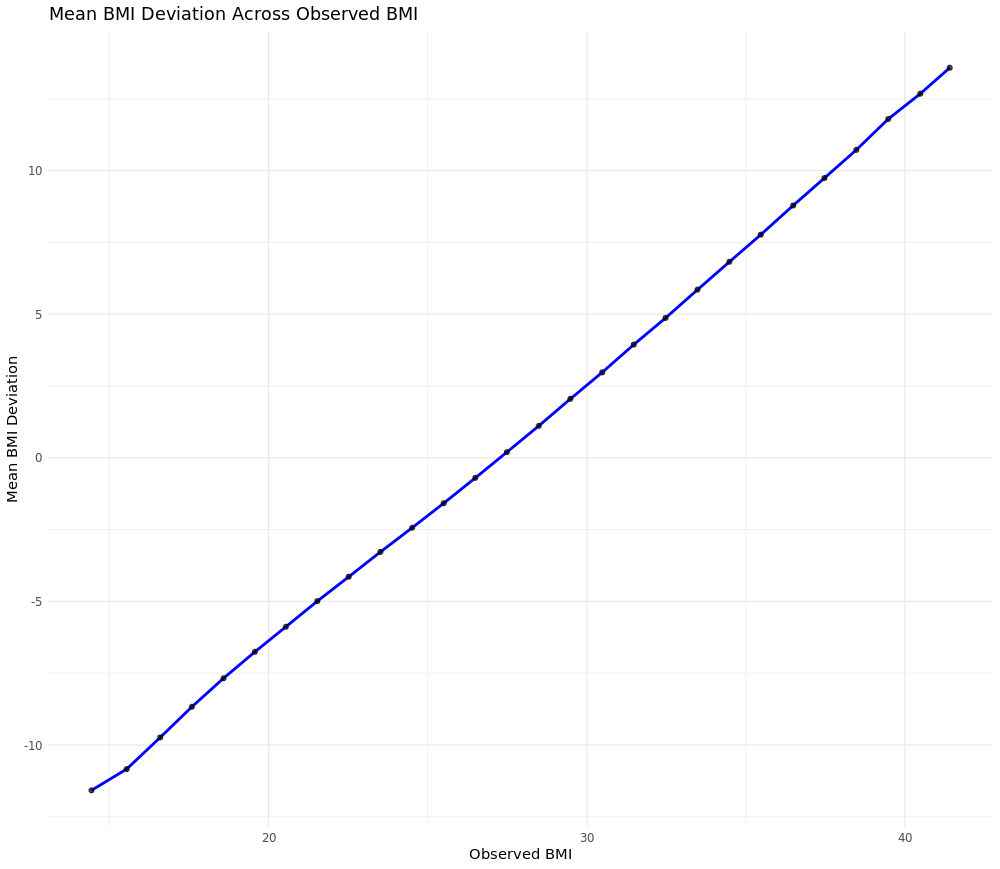


Supplementary Figure S2. Mean BMI deviation across observed BMI values. Participants were grouped into 1-unit observed BMI bins. Within each bin, we calculated the average deviation between observed BMI and genetically predicted BMI. The plot shows a strong positive relationship: individuals with higher observed BMI tend to have BMI values increasingly above their genetic expectation, while those with lower observed BMI tend to fall below.


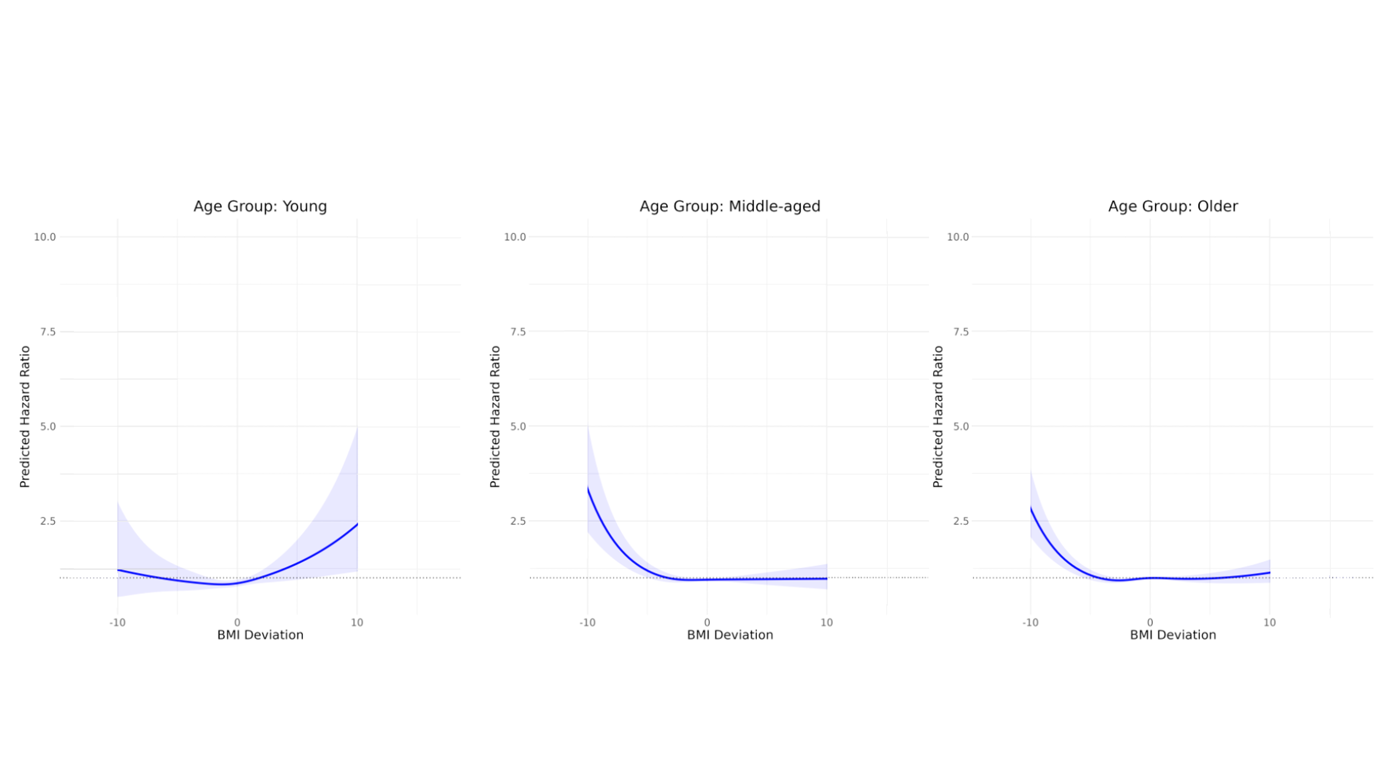


Supplementary Figure S3. Predicted Hazard Ratio as a Function of BMI Deviation, shown separately for individuals in age bracket 37-49 (left), 50-59 (middle), and 60-75 (right). Natural splines with four degrees of freedom were employed to model the non-linear effects of BMI deviation. The solid line represents the predicted hazard ratio across the range of BMI deviations, while the shaded area indicates the 95% confidence interval, highlighting the uncertainty around the predictions. The models were adjusted for sex, age at recruitment, total physical activity, Townsend Deprivation Index, aggregated income, and smoking category. The dotted horizontal lines indicate a hazard ratio (HR) of 1, highlighting the BMI at which mortality risk is equal to the reference group.


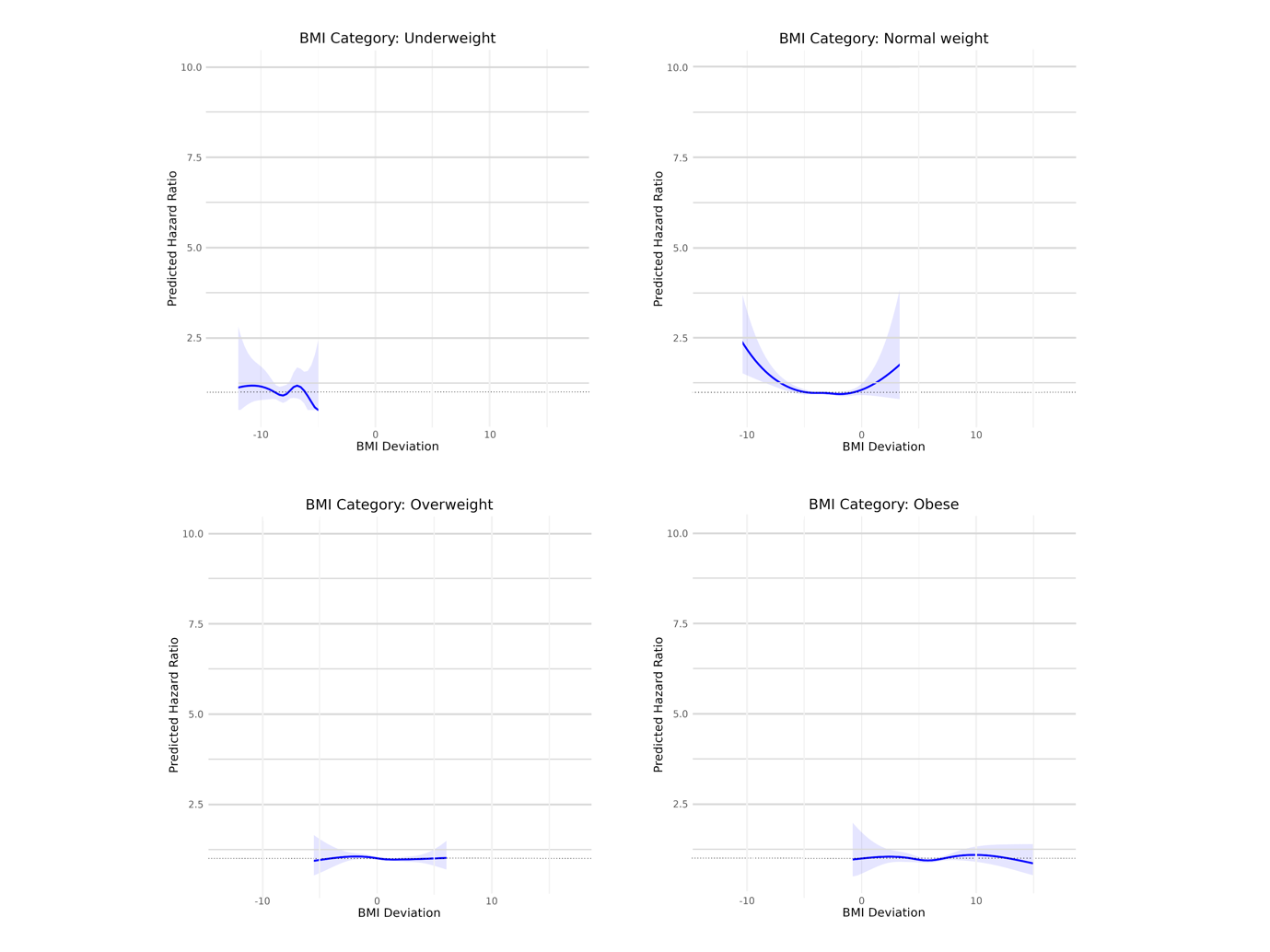


Supplementary Figure S4. Predicted Hazard Ratio as a Function of BMI Deviation, shown separately for individuals in different WHO-BMI categories: top-left, underweight; top-right, normal; down-left, overweight; down-right, obese. Natural splines with four degrees of freedom were employed to model the non-linear effects of BMI deviation. The solid line represents the predicted hazard ratio across the range of BMI deviations, while the shaded area indicates the 95% confidence interval, highlighting the uncertainty around the predictions. Extrapolated regions beyond the data range were removed for clarity. The models were adjusted for sex, age at recruitment, total physical activity, Townsend Deprivation Index, aggregated income, and smoking category. The dotted horizontal lines indicate a hazard ratio (HR) of 1, highlighting the BMI at which mortality risk is equal to the reference group.

## Supplementary Tables

Supplementary *Table S1. Distribution of Cause-specific Mortality in the UKB Cohort, Classified by Gender According to ICD-10 Codes During the Study Period*

| **Cause_of_death** | **Males (%)** | **Females (%)** |
| --- | --- | --- |
| Malignant neoplasms | 2794 (48.4%) | 2977 (51.6%) |
| Major cardiovascular diseases | 1037 (62.5%) | 623 (37.5%) |
| Diseases of respiratory system | 438 (57.7%) | 321 (42.3%) |
| Chronic liver diseases | 96 (73.3%) | 35 (26.7%) |
| Other disorders of circulatory system | 49 (55.1%) | 40 (44.9%) |
| Benign neoplasms | 43 (56.6%) | 33 (43.4%) |
| Infectious and parasitic diseases | 31 (42.5%) | 42 (57.5%) |
| Diabetes mellitus | 9 (90.0%) | 1 (10.0%) |
| Anaemias | 6 (60.0%) | 4 (40.0%) |
| Nutritional deficiencies | 0 (0.0%) | 2 (100.0%) |
| Other or unspecified | 1242 (53.7%) | 1070 (46.3%) |

## Supplementary Methods

Physical Activity (PA): Participants reported their weekly engagement in physical activities at different intensities: light, moderate, and vigorous. Each activity's duration was converted into minutes using predefined categories, with assigned values ranging from 0 minutes for 'None' to 360 minutes for '6+ hours' of activity per session. These values were summed to create a composite physical activity score for each participant, representing total weekly engagement in physical activities.

Socioeconomic Status (SES): SES was assessed using two primary measures:

Townsend Deprivation Score: Calculated from national census data prior to recruitment, this score integrates unemployment, car ownership, household overcrowding, and owner occupation rates to provide a measure of area-level socioeconomic deprivation. Higher scores indicate greater deprivation.

Annual Household Income: Self-reported income was categorized into economic brackets reflecting a spectrum from low to high socioeconomic status. The categories included less than £18,000; £18,000 to £30,999; £31,000 to £51,999; £52,000 to £100,000; and greater than £100,000. These categories were further aggregated into 'Low income', 'Middle income', 'High income', and 'Unknown income' for detailed analysis. Income data were transformed into dummy variables with 'High income' serving as the reference category to facilitate regression analysis. Smoking category: Categorized based on self-reports into 'Never', 'Former', and 'Current' smokers, providing insight into behavioral risk factors associated with various health outcomes.
